# The effect of socioeconomic status on severe traumatic injury: a statistical analysis

**DOI:** 10.1007/s00068-019-01219-w

**Published:** 2019-09-04

**Authors:** Zar Popal, Eva Berkeveld, Kees Jan Ponsen, Harold Goei, Frank W. Bloemers, Wietse P. Zuidema, Georgios F. Giannakopoulos

**Affiliations:** 1 Department of Trauma Surgery, Amsterdam University Medical Center (Amsterdam UMC, location VUmc), De Boelelaan 1117, 1081 HV Amsterdam, The Netherlands; 2 Department of Trauma Surgery, Northwest Clinics Alkmaar, Alkmaar, The Netherlands

**Keywords:** Socioeconomic status, Severe traumatic injury, Polytrauma

## Abstract

**Purpose:**

The amount of studies performed regarding a link between socioeconomic status (SES) and fatal outcome after traumatic injury is limited. Most research is focused on work-related injuries without taking other important characteristics into account. The aim of this study is to examine the association between SES and outcome after traumatic injury.

**Methods:**

The study involved polytrauma patients [Injury Severity Score (ISS) ≥ 16] admitted to the Amsterdam University Medical Center (location VUmc) and Northwest Clinics Alkmaar (level 1 trauma centers). The SES of every patient was based on their postal code and represented with a “status score”. Univariate and multivariable analyses were performed to estimate the association between SES and mortality, length of stay at the hospital and length of stay at the Intensive Care Unit (ICU). Z-statistics were used to determine the difference between the expected and actual survival, based on Trauma Revised Injury Severity Score (TRISS) and PSNL15 (probability of survival based on the Dutch population).

**Results:**

A total of 967 patients were included in this study. The lowest SES group was significantly associated with more penetrating injuries and a younger age (45 years versus 55 years). Additionally, severely injured patients with lower SES were noted to have a prolonged stay at the ICU. Furthermore, differences were found in the expected and observed survival, especially for the lower SES groups.

**Conclusion:**

Polytrauma patients with lower SES have more often penetrating injuries, are younger and have a longer stay at the ICU. No association was found between SES and length of hospital stay and neither between SES and mortality.

## Introduction

Trauma is one of the main causes of death in the Netherlands, especially in the younger population, and the number of trauma victims is increasing significantly. In 2015, more than 80,000 patients have been admitted to a Dutch hospital due to traumatic injury [1]. The majority of these injuries were related to fall accidents, road traffic accidents and suicide [2, 3]. Previous research suggested that there was an association between the social characteristics of the patients and their outcome after traumatic injury. For example, patients with a lower intelligence level, specific racial-ethnic background and no insurance policy had a higher risk on hospitalization and mortality due to trauma [4–10].

However, there is a scarcity of studies which examined the association between socioeconomic status (SES) and outcome after traumatic injury. Moreover, most research is focused on work-related injuries and does not take other important characteristics, like comorbidity of the patient, into account. In addition, nearly all reviews used only the education level to determine SES, whereas other indicators, such as household income and employment status, should be taken into consideration as well [11, 12].

The aim of this study is to examine whether there is an association between the SES and the clinical outcome of polytrauma patients after injury. Examining the association between SES and traumatic injuries can be useful for public health organizations in guiding their allocation of resources and to prioritize the implementation of preventative measures in specific neighborhoods.

## Methods

### Study design

A cross-sectional analysis was performed, including data from polytrauma patients [Injury Severity Score (ISS) ≥ 16] who were admitted to the level 1 trauma centers of the Amsterdam University Medical Center (Amsterdam UMC, location VUmc) and Northwest Clinics Alkmaar (NCA) between 1-1-2015 and 1-1-2018. Polytrauma patients were selected using the database of our trauma region (Network Acute Care Northwest), which is part of the National Trauma Registry (NTR) of the Netherlands. In the NTR, injuries are classified by the Abbreviated Injury Scale (AIS) 2005, update 2008 [13, 14]. All transport modes were included (ambulance, helicopter or self-referral). In addition, patients from all ages, gender and ethnicities are included.

### Data extraction

Patient characteristics, type of injury (blunt versus penetrating) and mechanism of injury (low and high energy falls, different types of road accidents, burning, shot or stabbing accidents and drowning) of each patient was extracted from the NTR. Mechanism of injury was reclassified into three main groups: traffic accidents (1), fall accidents (2) and others (3). In addition to the ISS and AIS, data concerning patients’ comorbidity status level were collected, including their Anesthesiologists Physical Status (ASA-PS) score [15, 16]. The ASA-PS classification system was used to determine the level of comorbidity based on the medical history before the time of injury. For this analysis, the variable was recoded into either a mild (ASA-PS ≤ 2) or severe (ASA-PS ≥ 3) comorbidity status before injury. Clinically relevant outcome measures included in-hospital mortality, length of stay at the hospital and at the ICU (in days).

### Socioeconomic status

Demographic and socioeconomic characteristics of the patients were determined using data from Statistics Netherlands (CBS) and The Dutch Institute for Social Research (SCP). The SES of every district was calculated and characterized by a ‘status score’, where a low status score represents a low SES. These scores included the following four indicators: the average income of people living within the district, the proportion of people with a lower level of education in the district, the proportion of people living with a low income and the proportion of unemployed residents. The study population was then divided into quantiles, based on the national SES [lowest (1)—highest (5)]. Postal code areas with less than 100 inhabitants were not included in this database to preserve the anonymity of the inhabitants and to exclude irrelevant areas such as industrial zones.

### Statistical analysis

Chi-squared analyses, one-way analyses of variance (ANOVA) and Kruskal–Wallis analyses were used for demographic and clinical characteristics. Multiple logistic regression and multiple linear regression were used to estimate the association between, respectively, SES and mortality and SES and (log-transformed) length of stay. Adjustments were made for known confounders, such as age, gender, comorbidity level, ISS, type, and mechanism of injury [17].

Additionally, a subanalysis for the probability of survival was performed to determine the difference between predicted mortality and actual mortality. This was determined using the Trauma and Injury Severity Score (TRISS) and the PSNL15 (Probability of survival specified for the Dutch population) [2, 18–21].

## Results

### Descriptives

A total of 967 patients were included in the study population, 676 patients from VUmc and 291 patients from NCA. Excluded were duplicates and patients with missing data. Missing data were mainly based on absent postal codes and status scores, as well as foreign patients. The study population consisted predominantly of male patients (68.9%) with a median ISS of 22 (range = 59). The five SES groups varied in size between 165 and 232 patients, with overall status scores ranging between − 3.42 and 2.53 (compared with a minimum of − 8.19 and a maximum of 2.93 in the national Dutch database). Most of the patients suffered from blunt trauma (96.2%), mainly due to traffic accidents (42.6%). An overview of baseline characteristics of each group can be found in Table 1.

**Table 1:** Patient and injury characteristics by socioeconomic status

Patient characteristics	Total population (*n* = 967)	Socioeconomic status					*p* value
		1 (lowest) *N* = 165 (17.1%)	2 *N* = 203 (21.0%)	3 *N* = 177 (18.3%)	4 *N* = 190 (19.6%)	5 (highest) *N* = 232 (24.0%)	
**Age**, mean (SD)*	52 (23)	45 (21)	50 (23)	56 (23)	53 (25)	55 (22)	< 0.001^b^
**Gender** male, *n* (%)	666 (68.9%)	119 (72.7%)	142 (70.4%)	118 (66.7%)	129 (67.9%)	155 (67.2%)	0.71^c^
**ISS**, median (range)	22 (16–75)	22 (16–75)	22 (16–50)	22 (16–59)	22 (16–75)	22 (16–75)	0.75^d^
**Comorbidity level**, *n* (%)^a^							
Severe (III–IV) (versus mild)	59 (6.1%)	8 (4.8%)	10 (4.9%)	14 (7.9%)	12 (6.3%)	15 (6.5%)	0.73^c^
Cause of injury*, *n* (%)							0.013^c^
Traffic accident	412 (42.6%)	41.8%	47.8%	36.2%	45.8%	50.9%	
Falling accident	376 (38.9%)	32.7%	31.5%	46.9%	39.5%	43.1%	
Other	179 (18.5%)	25.5%	20.7%	16.9%	14.7%	15.9%	
Type of injury*, *n* (%)							
Penetrating (versus blunt)	29 (3.8%)	14 (8.5%)	7 (3.4%)	7 (4.0%)	2 (1.1%)	7 (3.0%)	0.007^c^
**Mortality**, n (%)	230 (23.8%)	39 (23.6%)	41 (20.2%)	45 (25.4%)	47 (24.7%)	58 (25%)	0.74^c^

^a^Comorbidity level based on ASA-PS (comorbidity state before injury): mild (ASA-PS ≤ 2) versus severe (ASA-PS ≥ 3)

^b^One-way ANOVA

^c^Chi-squared analysis

^d^Kruskal–Wallis analysis

^*^Statistically significant (*p* < 0.05)

Univariate analyses showed that the lowest SES group (1) had severe traumatic injuries at a significantly younger age compared to the highest SES group (5) (45 years versus 55 years, *p* < 0.001). Furthermore, penetrating injuries occurred more frequently in the lowest SES group (1) (8.5% versus 3.0%, *p* = 0.007).

### Mortality

Initially, no significant association was found between SES and mortality (Table 1, *p* = 0.74). Table 2 shows that, after adding covariates to the model, there still was no association between the five SES groups and mortality. Only age, comorbidity level, mechanism of injury, ISS and AIS-head seemed to be significantly related to mortality (*p* < 0.05).

**Table 2:**
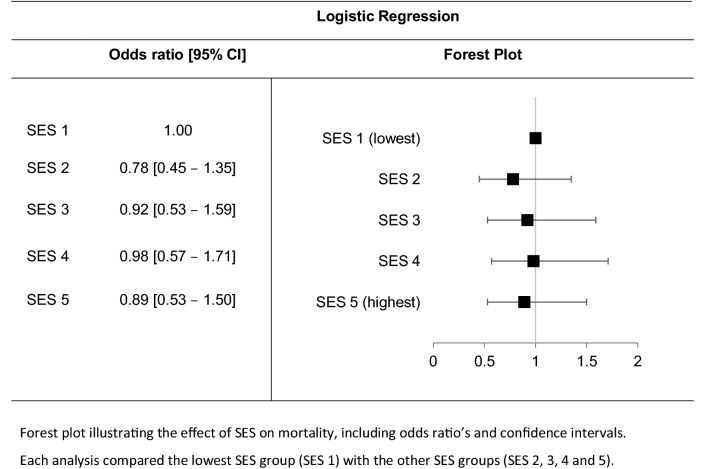
The effect of SES on mortality

### Length of stay

Length of stay at the ICU, however, was significantly related to SES (*p* = 0.04). Even after using a combined predictor model including SES, age, gender, comorbidity, type of injury, mechanism of injury, ISS and AIS, the results remained significant (*p* = 0.03). Length of stay at the hospital was not significantly associated with the SES groups (Table 3).

**Table 3:** The effect of SES on length of stay at the hospital and at the ICU

	Unstandardized coefficients	*p* value	[95% CI]
**Length of stay at hospital**			
Model 1	− 0.016	0.17	[− 0.04 to 0.01]
Model 2	− 0.021	0.08	[− 0.04 to 0.00]
**Length of stay at IC**			
Model 1*	− 0.020	0.04	[− 0.04 to 0.00]
Model 2*	− 0.020	0.03	[− 0.04 to 0.00]

Predictors model 1: socioeconomic status only

Predictors model 2: socioeconomic status, age, gender, comorbidity, type of injury, mechanism of injury, ISS and AIS

^*^Statistically significant (*p* < 0.05)

### Probability of survival

**Fig. 1:**
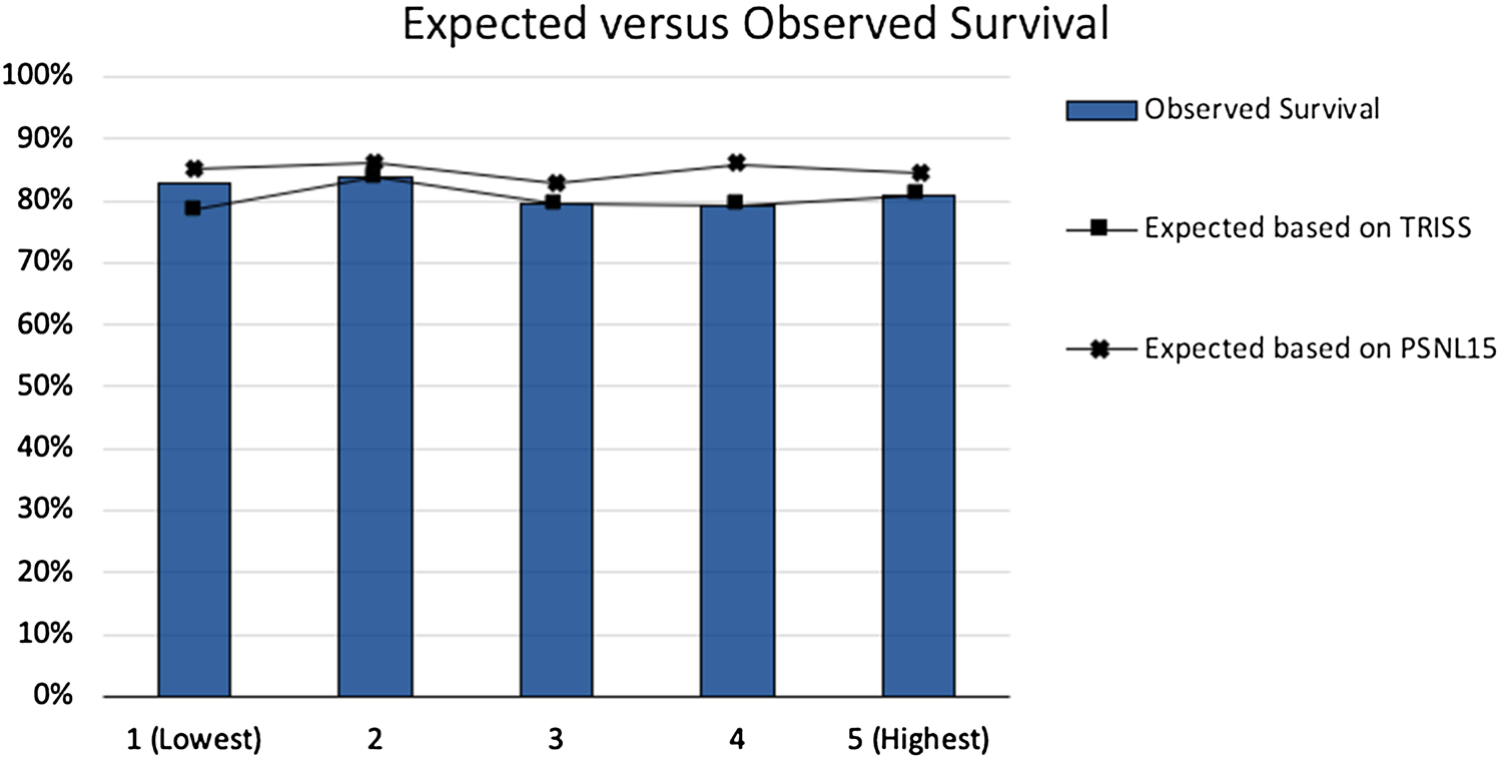
Differences between observed and expected survival

The subanalysis showed differences between expected survival (based on TRISS and PSNL15) and actual survival. Both the TRISS and the PSNL15 scores do not adequately correspond with the actual survival. The greatest difference between expected and observed survival can be seen in the lowest SES group (1). However, Z scores were only significant for SES 4. Details can be found in Table 4 and Fig. 1.

**Table 4:** Differences between observed and expected survival

	Socioeconomic status				
	1 (lowest)	2	3	4	5 (highest)
**TRISS**					
Expected survival	78.5%	80.9%	77.4%	80.5%	81.1%
Z score	− 1.68	− 1.25	− 0.81	0.58	0.03
**PSNL15**					
Expected survival	85.3%	86.0%	82.9%	85.9%	84.5%
Z score	1.02	0.98	1.37	2.96	1.66
**Observed (actual) survival**	82.9%	83.8%	79.5%	79.2%	81.0%

## Discussion

This analysis of polytrauma patients was performed to investigate a possible association between patients’ SES and clinical outcome after traumatic injury. We hypothesized that trauma patients with low SES levels were associated with worse clinical outcomes.

The analyses revealed that the lowest SES group seemed to include younger patients, with a doubled number of penetrating injuries compared to the highest SES group. However, in contrast with previous research in other countries, we could not find a significant association between socioeconomic status and mortality. Possible explanations include the following.

First of all, the geographical location of our trauma region was advantageous for this study, since it is considered as one of the most culturally diverse cities in the Netherlands. However, it is important to point out the current SES of the Dutch society. Indeed, there is a gap between the lowest and highest SES, but—compared to many other countries— this is quite small. Additionally, the social safety net of the Dutch government is actively supporting cases of socioeconomic deterioration, to promote equality within the society. Therefore, it is quite evident that we could not find any significant associations with our relatively homogeneous study population.

However, we did find a significant association between SES and length of stay at the ICU. Even though the association was minimal (unstandardized coefficient − 0.02), it is important to identify possible causes for these findings. As can be seen in Table 1, no significant differences were found in gender, ISS or comorbidity to explain these results. The combined predictor model, correcting for SES, age, gender, comorbidity, type of injury, mechanism of injury, ISS and AIS, still showed significant differences. Psychological factors in the lower SES groups, with more often mental stress and pressure to achieve due to work-related subjects, might have decreased their state of health in advance, resulting in a longer stay at the ICU [22]. However, additional analyses are recommended, for example regarding hypotension rate or amount of blood loss to eliminate or confirm these as possible confounders for our results.

Unexpected results were found in the subanalysis regarding the expected survival and the actual (observed) survival. The actual survival does not correspond with the predicted survival (based on TRISS) in the lowest SES group, with a difference of approximately 3–4% (Table 4, Fig. 1). This might suggest that other factors are required in analyses including lower SES groups. Furthermore, it is important to notice the more deviant PSNL15 scores, which do not match the TRISS nor the actual survival. Further adjustments for more accurate calculations can, therefore, be advisable.

Apart from that, there are limitations in our study that might (partially) explain the discrepancy in findings as well. One of them includes the difference in SES data that were used to examine the association. To improve the accuracy of the results, we used the database of StatLine Netherlands, which used four indicators to determine SES. However, the use of postal codes might have created risk of bias since the assumption of population homogeneity within a postal code is made, especially in emerging areas. Another limitation was the limited size of the included area. We were only able to obtain data from two level 1 trauma centers (Amsterdam UMC, location VUmc, and Northwest Clinics Alkmaar), whereas the inclusion of more hospitals in the Netherlands might have given a more accurate insight. Furthermore, our database did not include patients who died on scene due to their injuries. Exploring the proportion of patients who died on scene per socioeconomic status might provide additional insight in the association between SES and mortality. Finally, appropriate attention should be paid to the external validity of our results and how these can be extrapolated, given the uniqueness of our mixed study population.

## Conclusion

No direct association was found between SES and mortality. However, patients from lower SES suffered from severe injuries at a younger age, showed more penetrating injuries and a longer stay at the ICU. Additionally, discrepancies were found between the expected and actual survival. Therefore, additional research is recommended to find explanations for these findings and to create a more enhanced overview.

## Abbreviations

AIS: Abbreviated Injury Score
ASA-PS: American Society of Anesthesiologists Physical Status
CBS: Statistics Netherlands
GCS: Glasgow Coma Score
IC: Intensive care
ISS: Injury Severity Score
NTR: National Trauma Register
PSNL15: Probability of survival based on the Dutch population (2015)
RTS: Revised Trauma Scores
SCP: The Dutch Institute for Social Research
SES: Socioeconomic status
TRISS: Trauma Revised Injury Severity Score
